# Ferritins: furnishing proteins with iron

**DOI:** 10.1007/s00775-016-1336-0

**Published:** 2016-01-29

**Authors:** Justin M. Bradley, Nick E. Le Brun, Geoffrey R. Moore

**Affiliations:** Center for Molecular and Structural Biochemistry, School of Chemistry, University of East Anglia, Norwich Research Park, Norwich, NR4 7TJ UK

**Keywords:** Bacterioferritin, Ferritin, Ferroxidase, Iron oxidation, Iron storage

## Abstract

Ferritins are a superfamily of iron oxidation, storage and mineralization proteins found throughout the animal, plant, and microbial kingdoms. The majority of ferritins consist of 24 subunits that individually fold into 4-α-helix bundles and assemble in a highly symmetric manner to form an approximately spherical protein coat around a central cavity into which an iron-containing mineral can be formed. Channels through the coat at inter-subunit contact points facilitate passage of iron ions to and from the central cavity, and intrasubunit catalytic sites, called ferroxidase centers, drive Fe^2+^ oxidation and O_2_ reduction. Though the different members of the superfamily share a common structure, there is often little amino acid sequence identity between them. Even where there is a high degree of sequence identity between two ferritins there can be major differences in how the proteins handle iron. In this review we describe some of the important structural features of ferritins and their mineralized iron cores, consider how iron might be released from ferritins, and examine in detail how three selected ferritins oxidise Fe^2+^ to explore the mechanistic variations that exist amongst ferritins. We suggest that the mechanistic differences reflect differing evolutionary pressures on amino acid sequences, and that these differing pressures are a consequence of different primary functions for different ferritins.

## Introduction

**Fig. 1:**
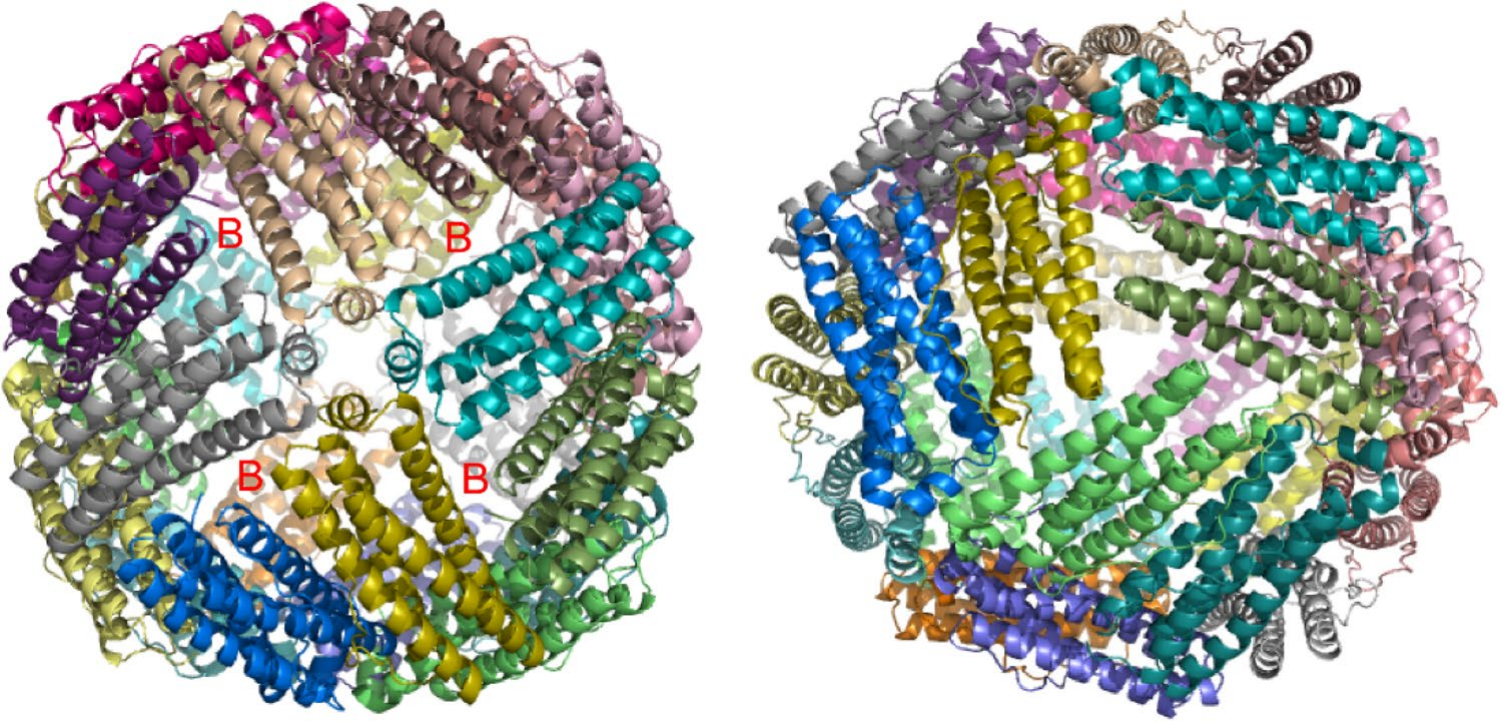
Overall structure of 24meric ferritins. *Left*, view down one of six fourfold channels through the protein coat. The locations of four of the 24 B-channels are indicated by ‘B’. *Right*, view down one of eight threefold channels. Generated using PyMol with PDB file 1BCF

Ferritins are characterized by their ability to accumulate large deposits of non-heme iron. Laufberger [1] coined the name ferritin in 1937, from the Latin *ferratus* (meaning furnished, covered or shod with iron [2]), to describe the iron-rich protein he had crystallized. Now the name ferritin is used to describe a superfamily of proteins as well as the specific type of protein exemplified by animal ferritins [3–10]. Most members of the ferritin superfamily consist of 24 subunits arranged to form an approximately spherical protein shell into which non-heme iron is deposited (Fig. 1). A single ferritin molecule of this type can hold up to 4300 iron ions in its central cavity [3, 11]. In addition to the 24-mer ferritins, so-called mini-ferritins composed of 12 subunits have been discovered in bacteria that can accommodate much smaller amounts of iron in their smaller central cavities [6]. Almost from the first description of ferritin up to the present time, the mechanism(s) by which such proteins accumulate iron has been intensively studied and yet despite the huge volume of work reported a full description of how any ferritin operates has not yet been achieved. The original publication by Granick and Michaelis in 1943 on the preparation of apo-ferritin [12], protein in which the non-heme iron has been removed, established that simple procedures with Fe^3+^ salts failed to reconstitute holoferritin, the iron mineral-containing form, and since then a variety of in vitro methods for preparing holoferritin have been described. However, as with much of the literature on the mechanism(s) of ferritin activity, it is not clear that any of the described procedures mimic exactly physiologically relevant mechanisms. In large part this lack of clarity stems from the complex chemistry of one of the ferritin substrates, iron. Fe^3+^ ions are poorly soluble in aqueous solutions and Fe^2+^ ions are susceptible to oxidation in aerobic environments. Presumably, this complex chemistry is the reason ferritins exist; it is important for biological organisms to sequester excess iron in a manner that does not lead to insoluble aggregates of Fe^3+^ ions interfering with their normal biochemistry. Putting the iron into a protein shell is an elegant solution. This review discusses how selected 24mer ferritins accumulate iron in vitro. The chosen proteins have been selected either with a view to exploring their physiological roles, and where possible we highlight the connections between these and the in vitro data, or because they illustrate a striking difference in behavior to related proteins despite similar structures.

**Fig. 2:**
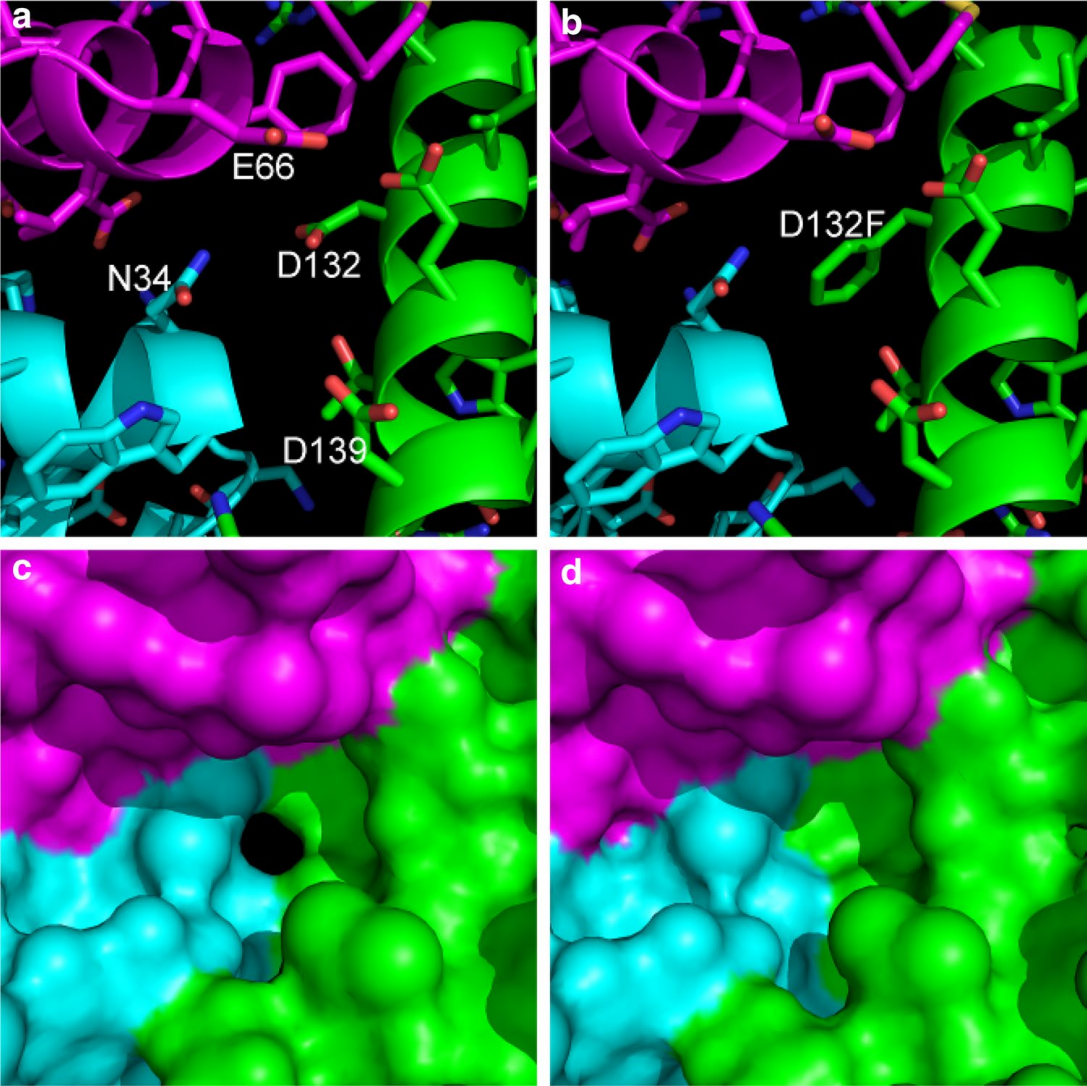
Comparison of B-channels in wild-type *E. coli* BFR and its D132F variant. One of the B-type channels formed at the interface between three subunits is displayed with the separate subunits coloured *magenta*, *cyan* and *green*. The amino acids forming the B-channels and the molecular surfaces generated with a 0.8 Å solvent probe radius (to mimic the hydrated Fe^2+^ substrate) are displayed for wild-type BFR (**a**, **c**) and its D132F variant (**b**, **d**). The D132F variant was constructed along with other variants involving residues lining the B channels to investigate whether the B channels are important for iron core formation [16] Constructed from PDB 1D3E1L. Reproduced with permission from Wong et al. [16]

24-mer ferritins share a common structure (Fig. 1): a protein shell assembled from 24 4-α-helical bundle subunits surrounding an internal cavity of ~80 Å diameter; channels through the protein coat that connects the inner cavity with the protein’s external environment; and catalytic sites that promote the oxidation of Fe^2+^ to Fe^3+^. There are major differences as well, most notably that the bacterioferritin (BFR) family of proteins contains up to 12 *b*–type heme groups at intersubunit sites, while the other 24mer ferritins are heme-free [5, 13, 14]. There are also differences in the number and types of channels through the shell. All 24mer ferritins have channels at the threefold and fourfold symmetry axes, but BFRs and heme-free prokaryotic ferritins (FTNs) also have an additional set of B-channels [15] which are not aligned with an axis of symmetry and occur where three subunits interact (Fig. 2).

The nature of the catalytic sites for Fe^2+^ oxidation, known as the ferroxidase centers and located in the middle of each subunit, is different as well [9, 17–25]. Though ferroxidase centers were first identified in human ferritin and BFR more than 20 years ago, the differences in their properties has meant that even today it is doubtful that all the key structural influences on the centers have been identified. This is an important point because these differences go to the heart of different interpretations of mechanistic data. Later we shall describe in detail the ferroxidase centers of our selected ferritins to illustrate this. Despite the high degree of structural similarity between ferritins, there is relatively little amino acid sequence identity between some members of the family, for example, BFR and FTN, beyond conservation of many of the residues forming the ferroxidase centers [7].

At a time when so many of the reported studies on ferritins involve the use of proteins prepared by recombinant methods, it is relevant to note that it is not only the protein shells of ferritins that differ, but also the nature of the iron cores they have accumulated in their central cavities. Generally, recombinant ferritins are isolated with relatively little iron content whereas native ferritins are often heavily iron-loaded, with the majority of the iron contained in a mineralized form. Native animal ferritins are generally reported to contain Fe^3+^ poly-oxo-hydroxide cores that are highly crystalline while plant ferritins and BFRs usually have phosphate-rich Fe^3+^ cores with an amorphous morphology. We return to this topic later.

In vitro mechanistic studies on the uptake of iron by ferritins has concentrated almost exclusively on the reaction of ferritins with added Fe^2+^ ions in the presence of an oxidant, most usually O_2_. In the absence of a ferritin the aerobic oxidation of Fe^2+^ below pH ~7 is rather slow. Ferritins catalyze oxidation of Fe^2+^ by O_2_ but to do so the Fe^2+^ ions have to enter the protein shell where the Fe^3+^ ions produced are trapped within the protein. There are many questions that need to be considered to describe this process, including:

1. How does Fe^2+^ enter the protein shell?
2. Where does the Fe^2+^ get oxidized?
3. How does the cavity accumulate Fe^3+^?

These are overarching questions and, as we shall see, some of the issues at the forefront of ferritin research concern detailed chemical questions. For example, at least some of the Fe^2+^ gets oxidised at ferroxidase centers and how the di-iron site is formed and whether it breaks down following Fe^2+^ oxidation are major concerns (see, for example, [9, 26–28]). A current issue in ferritin research is whether there is a universal mechanism that accounts for core formation resulting from the aerobic oxidation of Fe^2+^, as Hagen and his colleagues have proposed [26]. Elsewhere we have set out some of the reasons why we believe a universal mechanism does not exist [9] and we will not address this issue directly here. However, it should be clear from our later discussion that the ferritins we have selected for detailed consideration do not follow the same mechanistic pathway and therefore a universal mechanism cannot exist. An additional important mechanistic issue that we have referred to above is that it is unclear whether the in vitro procedures used to study core formation in ferritins are always physiologically relevant. This is not least because most supply iron to the ferritin in the form of hydrated Fe^2+^ and it seems likely that within cells the available Fe^2+^ is bound to ligands, some of which may act as the iron donors to ferritins. As Philpott notes [29], “the iron chaperone field is in its infancy, with many more questions unanswered than resolved”, but iron donors for ferritins have been identified [29, 30]. Leaving aside the issue of the supply of Fe^2+^ to ferritin for now, we believe the mechanistic studies of the aerobic uptake and oxidation of Fe^2+^ by ferritins is physiologically relevant where O_2_ is the natural oxidant.

In the 79 years since Laufberger first described ferritin [1] what must have struck most chemists encountering it for the first time is the fact that as much as 24 % of the protein by weight may be Fe^3+^ and yet it is highly soluble in water at pH 7. For many of us this is the central feature of ferritins, and is an observation that probably accounts for the description of ferritin as an iron-storage protein. However, iron-storage is a functional term. Is the primary function of all ferritins iron storage? Probably not! Andrews [7], Theil [8] and Arosio et al. [10] have considered the functional aspects of ferritins in recent reviews and they provide impressively long lists of physiological functions in addition to iron trafficking and storage. Most of the additional functions they list though can be reduced to the chemical property of being an antioxidant. Indeed, it is clear that the genes encoding some prokaryotic ferritins are only induced under conditions of oxidative stress (see, for example, [31, 32]). Whether this is because the ferritin that is upregulated reduces oxidative stress by catalyzing the removal of O_2_ or reactive oxygen species as Fe^3+^ is produced from an Fe^2+^ substrate, removing excess Fe^2+^ ions, or providing a source of iron for the production of enzymes such as catalase, which directly reduces oxidative stress by disproportionating H_2_O_2_, has not always been demonstrated but the role in reducing oxidative stress is clear. Steifel and Watt [13], noting the redox activity of Fe^3+^ core formation from Fe^2+^, suggested that one of the roles of BFR was as an electron source, something that might fit in with the earlier description of BFR as a cytochrome [13, 33]. The precise physiological functions of ferritins is an important topic in its own right, but it is also important in terms of considering mechanisms of iron core formation in ferritins in vitro. The evolutionary pressures on proteins are such that provided the protein can perform its function satisfactorily mutations can be accepted [34]. This means, for example, that if the main function of a ferritin is relief of oxidative stress by the rapid removal of O_2_, the key residues involved in this might be more resistant to mutation than the corresponding residues of a ferritin whose main role is to sequester excess iron in a form that can be rapidly mobilised when the cell requires it, assuming, of course, that the key residues for these two activities are not the same. As we consider individual ferritins below, the issue of the physiological relevance should be borne in mind.

In addition to considering how ferritins build up a core in vitro we briefly describe recent advances in determining how ferritins release iron. The release of iron by ferritins is not as well understood as their uptake of iron but it is an important subject for those ferritins that are not dead-end iron stores. Though iron release is not well described yet, as we shall see it is already apparent that there is great diversity in physiological iron release processes. Thus, just as a universal mechanism does not exist for iron uptake nor is there a universdal mechanism of iron release.

## The iron cores of ferritins

If ferritins are primarily iron-storage proteins then what form they store the iron in becomes an important subject, as this will be the end product of storage and, perhaps, the starting point for iron release. However, if the primary function of a ferritin is something other than iron storage then the nature of the iron core may not be so important. Unfortunately, we usually don’t know what the primary function of a ferritin is, so are not sure how important knowledge of its native iron core is. This uncertainty, coupled with the current prevalence of studying recombinant proteins, means that the form of the stored iron in most native ferritins has not received much attention in the past 30 years. Because of this we shall briefly review the earlier literature on the cores of native ferritins as well as consider more recent studies on human ferritin. Before doing so we should note that for all ferritins, the nature of the core appears to be influenced more by the environment the ferritin is in than the protein coat itself. This is evident from the studies of ferritin cores reconstituted in vitro compared to the native cores they had on isolation that are described below. In this context, an important point to note is that some of the studies of native human ferritins have been with samples from patients suffering with a variety of diseases that affect iron metabolism, such as beta-thalassemia [35] or haemochromatosis [36], and whether the molecular details of the native ferritin cores found in these studies can be extrapolated to the ferritins from humans not suffering from such a disease is not clear, particularly since the flow of iron into the ferritins is likely to be different under normal and iron overload conditions. Nevertheless, such studies are revealing and we have referred to some below.

### Heterogeneity and maximum iron content of core

Native ferritins are heterogeneous because though the protein shells of a pure ferritin may be homogeneous the iron cores are not [3, 37]. This is a major complication for many physical techniques. For example, it is largely because of this that X-ray structures of ferritins containing a core have not revealed what the core looks like at atomic resolution: the crystals have regularly packed protein shells whose diffraction patterns yield their structure, while the irregularly packed cores do not. Fischbach and Anderegg [11] used isopycnic centrifugation to separate horse spleen ferritin into relatively homogeneous fractions as determined by size, thus partially overcoming the problem of heterogeneity, and found that what they called “full” ferritin had a molecular mass of ~900 kDa and contained ~4300 Fe^3+^ ions per molecule. However, even though the cores of ‘full” ferritin were about the same size they were irregularly packed in crystals, which still prevented their structures from being determined by X-ray diffraction [38].

### Composition

The composition of native cores varies considerably (Table 1), with some being phosphate-rich. We have not been selective in our tabulation of data for the cores of BFR and FTN as, apart from those listed in Table 1, we are not aware of additional relevant studies of these. Therefore, although the literature on in vitro mechanistic studies of *E. coli* FTN and BFR is extensive, there are no reports of the characteristics of their native cores beyond the early description [55] of the ^57^Fe Mössbauer spectrum of *E. coli* cells loaded with ^57^Fe which identified the spectroscopic signature of a polynuclear iron species that had the characteristics of an amorphous material, assumed to be the BFR subsequently isolated from such cells, and a later ^57^Fe Mössbauer study [56] of wild-type, *bfr*^−^ and *ftn*^−^ cells which showed the FTN to have a magnetically ordered core, and thus must at least have had limited crystallinity.

**Table 1:** Characteristics of iron cores of native ferritins

Ferritin^a^	Number of iron ions	Number of phosphates	Fe:P ratio	Core morphology^b^	Refs.
Animal ferritins	See footnote ^c^				
Human spleen ferritin	2200	105	21:1	C	[39]
Chiton (*Acanthopfeura* *hirtosa*)	1500–2500	40–70	36:1	L	[40]
Limpet (*Patella laticostata*)	2000	45	44:1	L	[40]
Pea phytoferritin	1800	640	2.8:1	A	[41]
Clover phytoferritin	1300	325	4:1	n.d.	[42]
*Helicobacter pylori* FTN	900	640	1.4:1	n.d	[43]
*Azotobacter vinelandii* BFR	600–1000 (mid)^d^ 1200–2000 (stat)^d^	430–715 (mid)^d^ 860–1430 (stat)^d^	1.4:1	A	[13, 44]
*Rhodobacter capsulatus* BFR	900-1000	600	~1.6:1	A	[45]
*Pseudomonas aeruginosa* BFR/FTN^e^	700	410	1.7:1	A	[46–48]

^a^The entries in this table are for the ‘as-prepared’ heterogeneous ferritins and thus the iron and phosphate contents represent an average

^b^ *C* crystalline, *L* limited crystallinity, *A* amorphous, *n.d.* not determined

^c^Native animal ferritins have been isolated from a wide variety of sources with iron contents from negligible to full loading but often the core morphologies of the samples were not reported, probably because EM facilities were not as available as now. Typical early examples are: horse spleen ferritin with an average iron content of 20–23 % and phosphate content of 1.5–2.0 % which was homogeneous by electrophoresis (i.e. had a uniform protein shell) but inhomogeneous in the ultracentrifuge (i.e. had a variable core size) [37], and ferritin from tadpole red blood cells with an average iron content of 12 % and a range of 8–24 % [49]. We have only cited a selection of studies in the table where a full analysis of core composition, size and morphology was reported. Chasteen and Harrison [50] provide further examples

^d^mid = cells harvested in mid-exponential phase; stat = cells harvested in stationary phase

^e^The *P. aeruginosa* samples used in these studies were mixtures of BFR and FTN (see text) although this was not known at the time and it was thought [51] the two types of subunit detected formed a BFR heteropolymer similar to the H/L heteropolymers of animal ferritins [3, 8] (see text). An example of the SDS-PAGE gels for a typical preparation of the *P. aeruginosa* samples is Fig. 4 of al-Massed et al. [52]. Importantly, however, the ^57^Fe Mössbauer spectra for samples of purified *P. aeruginosa* ferritins and intact cells [53] over a wide temperature range revealed only one type of core suggesting that both its BFR and FTN have similar amorphous phosphate-rich cores. All these studies were carried out with cells grown on a high nitrate medium. On a low nitrate medium ^57^Fe Mössbauer spectra of *P. aeruginosa* cells contained two signals, one likely from the kind of amorphous core observed in the studies described above and a signal from a more ordered core which likely contained regions of limited crystallinity [54]. As far as we aware isolation of ferritins from such cells has not been reported so it is not known whether the more ordered core comes from BFR or FTN

There is a significant difference between some ferritins in their phosphate contents beyond the relative amounts. The phosphate of ferritins with a low Fe:P ratio is incorporated into the core in a way that means it is not removed without loss of iron, whilst the phosphate of the ferritins with high Fe:P ratio is relatively easy to remove and does not affect the structure of the core iron. This is consistent with a model of the core for the low phosphate containing ferritins being an Fe^3+^ poly-oxo-hydroxide polymer with phosphate associated with its surface, and thus close to the inner surface of the ferritin protein shell, rather than integrated into the mineralized iron core as is the case with the phosphate-rich cores [3, 57, 58].

### Morphology

The morphology of native cores varies considerably, with some being highly crystalline, others being highly amorphous and others being somewhere between the two extremes (Table 1). EM [39, 57, 59] and ^57^Fe Mössbauer spectroscopy [40, 47, 60] have been the main methods for determining core morphologies, with both being applicable to ferritins in situ in biological samples as well as when purified. EM allows the crystallinity of cores to be characterized directly while ^57^Fe Mössbauer spectroscopy over the temperature range 200–1.3 K allows the intrinsic magnetic properties of the core to be studied, which report on the long-range order within the core.

The size distributions of cores determined by EM have been widely reported. For as-prepared samples that have not undergone fractionation there is usually a large range of sizes. Examples are: horse spleen ferritin with a core size distribution having diameters from 24 to 78 Å and a median value of 50–60 Å [57] and *P. aeruginosa* BFR/FTN (see note 5 to Table 1) from 40 to 80 Å and with a median value of 60–65 Å [46]. A fraction of the horse spleen ferritin studied by Mann et al. [57] containing ~3000 iron ions per molecule had a more homogeneous distribution but its core diameter still varied over the range 30–70 Å. Clearly, with the considerably different amounts of iron in the cores of horse spleen ferritin and the *P. aeruginosa* samples, the similarity in their core sizes means the cores of the *P. aeruginosa* ferritin samples were much less dense than the cores of horse spleen ferritin. This is consistent with the phosphate being an integral part of the core, something confirmed by EXAFS studies of *A. vinelandii* BFR [58]. Electron diffraction patterns for horse spleen ferritin and human ferritins measured by EM are indicative of the core having a structure resembling that of ferrihydrite (see below). Electron diffraction for *A. vinelandii* BFR and *P. aeruginosa* ferritin samples showed no evidence of crystallinity [57] although some preparations of native *A. vinelandii* BFR have been reported to contain small regions of crystalline ferrihydrite within their otherwise amorphous phosphate-rich cores [44].

Since polynuclear clusters of Fe^3+^ contain antiferromagnetically coupled high-spin Fe^3+^ ions, their ^57^Fe Mössbauer spectra can be temperature dependent because their magnetic properties are affected by the variation in thermal energy [47, 60]. An important phenomenon for interpreting ferritin ^57^Fe Mössbauer spectra is superparamagnetism. This occurs in magnetically ordered materials where transitions of the sublattice magnetizations between energetically equivalent crystallographic directions are thermally activated. The total electron spin associated with the net magnetic moment of a ferritin core is confined to certain energy levels corresponding to allowed orientations of the spin with respect to the axis of quantization. The ground state can adopt one of two orientations, the so-called ‘easy’ directions, which are equal in energy and correspond to spin up and spin down. These two ‘easy’ directions are separated by an energetic barrier, or ‘hard’ direction. At temperatures where there is sufficient thermal energy to allow the spins to surmount the hard barrier the spins can flip orientation and the ^57^Fe Mössbauer spectrum is a quadrupole-split doublet. At lower temperatures where there is insufficient thermal energy to allow the spins to overcome the barrier the ^57^Fe Mössbauer spectrum is magnetically split and becomes a sextet. As samples are usually heterogeneous there is a range of core sizes with differing magnetic properties so that the doublet and sextet components co-exist in the spectrum. The average blocking temperature of a sample is usually considered to be the temperature at which 50 % of the ^57^Fe Mössbauer spectrum is the magnetically split sextuplet signal and 50 % the quadrupolar split doublet. Increasing crystallinity of the core is associated with higher blocking temperatures. On this measure, horse spleen ferritin and human ferritin having much higher blocking temperatures than *A. vinelandii* BFR and *P. aeruginosa* BFR/FTN (see note 5 to Table 1) and are considered [44, 47] to be more ordered than the bacterial ferritins, in keeping with the EM studies referred to above.

EPR [61] and magnetic circular dichroism [62] spectroscopies also reveal differences between crystalline and amorphous cores of ferritins, with the theoretical understanding of the EPR spectrum of crystalline cores sufficiently developed to allow the blocking temperature to be determined. As Wajnberg et al. [61] note, the blocking temperature determined by EPR spectroscopy is different from that measured by ^57^Fe Mössbauer spectroscopy because the two methods have different measurement times and they describe a procedure that allows the two values to be compared taking into account this variable.

### Structures of crystalline cores

Harrison and her co-workers noted that the X-ray diffraction pattern of the crystalline cores of human and horse spleen ferritins resembled those for the mineral ferrihydrite [63], and ever since then successive studies with a variety of techniques have reaffirmed the view that ferrihydrite is the dominant component of crystalline cores [3, 8]. Ferrihydrite has the chemical composition 5Fe_2_O_3_.9H_2_O, and recently Sadeghi et al. [64] have demonstrated that the aqueous chemistry of Fe^3+^ ions in the absence of ferritins generates an Fe_13_ polymer that resembles ferrihydrite. This observation is consistent with the view that ferritins provide an enclosed reaction vessel in which an iron-rich core can be laid down rather than a surface that catalyzes formation of a particular kind of iron-containing polymer.

Pan et al. [36] reported that scanning transmission electron microscopy of ferritin in liver biopsy samples from a patient suffering with type 2 hereditary haemochromatosis, which contained 200–4000 iron ions per ferritin molecule, suggested that their cores were composed of ferrihydrite contained in eight core particles with an arrangement reflecting the cubic symmetry of the protein shell so that the core particles were associated with the eight threefold symmetry channels. This extends considerably the description of the particle structure of the core first discussed in earlier publications using EM, which were based on four or six components. The association of the core particles with the threefold symmetry channels allowed Pan et al. [36] to propose a scheme for core formation in human ferritin based on Fe^2+^ entering the central cavity via the threefold channels. Their model is consistent with other data that shows Fe^2+^ ions enters the central cavity via the threefold channels [4, 8]. Theil and her colleagues provide compelling evidence [20, 65] on this point by observing metal ions in transit through the protein coat with X-ray crystallography. They show a line of metal ions, 15 Å long, that spans the protein coat through the through-fold channels, and note that each Fe^2+^ ion travels a distance of 28 Å through the protein to reach the ferroxidase centers. However, Pan et al. [36] suggest the bulk of the Fe^2+^ ions gets oxidized in the cavity whilst the scheme that Theil and her colleagues have developed has it being oxidized at the ferroxidase centers. It remains to be seen whether this is a consequence of Pan et al. [36] studying ferritin formed under high iron conditions that overwhelm the relatively few ferroxidase centers in the molecule, or whether the core structure Pan et al. [36] observe can be formed by the oxidation and transit pathway in the Theil model [20, 65]. What is clear is that the experimental data used to identify the site of oxidation in the Theil model is extensive and convincing while Pan et al. [36] did not provide independent experimental data to identify the site of oxidation in their model.

### Influence of the protein shell on characteristics of the core

An interesting question arising from these observations that has occupied many researchers in the field, is why are some cores highly crystalline and others amorphous? It is well established that it is not the protein shell that is responsible since reconstituted cores of bacterioferritins in vitro can be well-ordered (for example, [56, 57]). Nor is the presence of large amounts of phosphate in the core a necessary requirement since the low-phosphate cores of molluscan ferritins have limited crystallinity (Table 1), and the phosphate-rich core of *A. vinelandii* BFR, though described as amorphous in most reports [57], has regions of limited crystallinity [44]. Generally, authors of studies addressing this issue suggest it is the rate at which cores are formed that determines their degree of ordering, or a combination of the rate and the amount of ligands other than hydroxide and oxide that is available to the newly formed Fe^3+^ ions that is important. A faster rate is taken to lead to a less ordered core. Thus the protein shell does not influence directly core morphology, and nor does it influence the size of the core. As Theil [66] notes, mineral sizes are relatively independent of constraints imposed by the protein shell and reflect iron bioavailablity for mineralization that is normally far below the maximum capacity.

However, an important feature of animal ferritins illustrates that the protein shell can influence the characteristics of the core. Animal ferritins are generally composed of two type of subunit, H for Heavy and L for Light, that coassemble to form heteropolymers with the relative numbers of H and L chains in a 24mer heteropolymer being tissue-specific [3, 8]. The properties of the H and L subunits are different, with only the H chain containing a ferroxidase center, and this means that the rate at which heteroploymers can accumulate iron varies [67, 68]. This, in turn, seems to correlate with characteristics of the core since different heteropolymers isolated from different tissues have different crystallinities and shapes [35, 69].

## Studies of native bacterioferritins

Bacterioferritins have been identified in numerous species, but studies of the native iron-loaded forms are not that extensive. Steifel and Watt [13] were the first to show that native BFR had a different mineral core to mammalian ferritins by reporting that *Azotobacter vinelandii* BFR contained a phosphate-rich core with an Fe:P ratio of 1.4:1. Subsequently it was reported that the native BFRs of *Pseudomonas aeruginosa* and *Rhodobacter capsulatus* also had phosphate-rich cores. The use of ^57^Fe Mössbauer spectroscopy to study BFRs has been particularly revealing since it is a method that can be applied to intact cells. In the case of *P. aeruginosa,*^57^Fe Mössbauer spectra of intact cells grown to stationary phase on media enriched with ^57^Fe were similar to those of purified native BFR [53]. Though it was known at the time of the ^57^Fe Mössbauer study that *P. aeruginosa* had at least two ferritin genes and the polypeptides from both were present in the cells, it was not known whether they formed homopolymers or a heteropolymer. Since then it has been established that the *bfrA* gene actually codes for a FTN [70], and thus might more properly be called *ftnA*. The two genes are regulated by iron differently, with *ftnA* (previously *bfrA*) constitutively expressed during the exponential growth phase and iron regulated on transition into the stationary phase, while *bfrB* is strongly upregulated under high-iron conditions [32, 71]. This has led on to detailed studies in vitro of the BFR B homopolymer (see below). What it means for the ^57^Fe Mössbauer spectra of intact cells [53] from which the ferritin studied by Moore and his colleagues was isolated [46, 51] is that the spectra reported are likely to be from a mixture of BFR B and FTN A (see note 5 to Table 1), with both having similar amorphous cores, or the FTN not having a sufficiently large core to give a detectable magnetically ordered spectrum indicative of at least limited crystallinity, as observed for other FTNs [43, 56]. The difference in genetic regulation of the two *P. aeruginosa* ferritin genes is consistent with FTN A being the general housekeeping ferritin for iron storage and BFR B being involved in relief of oxidative stress [32, 71].

^57^Fe Mössbauer spectra of intact *Rhodobacter capsulatus* cells have also been reported [45]. *R. capsulatus* is a purple photosynthetic bacterium able to grow aerobically and anaerobically (photosynthetically). The native BFR isolated from aerobically grown cells contained an amorphous core of 900–1000 iron ions per 24mer with an Fe:P ratio of 1.5–1.7:1. The iron in the BFR cores in both aerobically and anaerobically grown intact cells was largely Fe^3+^ which raises the issue of what the oxidant for Fe^2+^ is in the anaerobically grown cells. The amount of BFR protein per cell was found to vary with iron content in the medium, being low when the medium was low in iron and high when the medium was enriched with iron, consistent with this BFR having a role in iron metabolism.

Ironically given that *E. coli* BFR is the best characterised BFR in vitro, its physiological role is not certain [56], though likely to be connected with control of oxidative stress as with other BFRs. From growth and ^57^Fe Mössbauer studies [56] of wild-type, *ftn*^−^ and *bfr*^−^*E. coli* strains FTN appears to fulfill the general iron housekeeping role, since the ^57^Fe Mössbauer spectra of wild-type cells grown to stationary phase reveal that the majority of the iron present is in FTN.

## Mechanistic studies of *Escherichia coli* bacterioferritin (EcBFR)

**Fig. 3:**
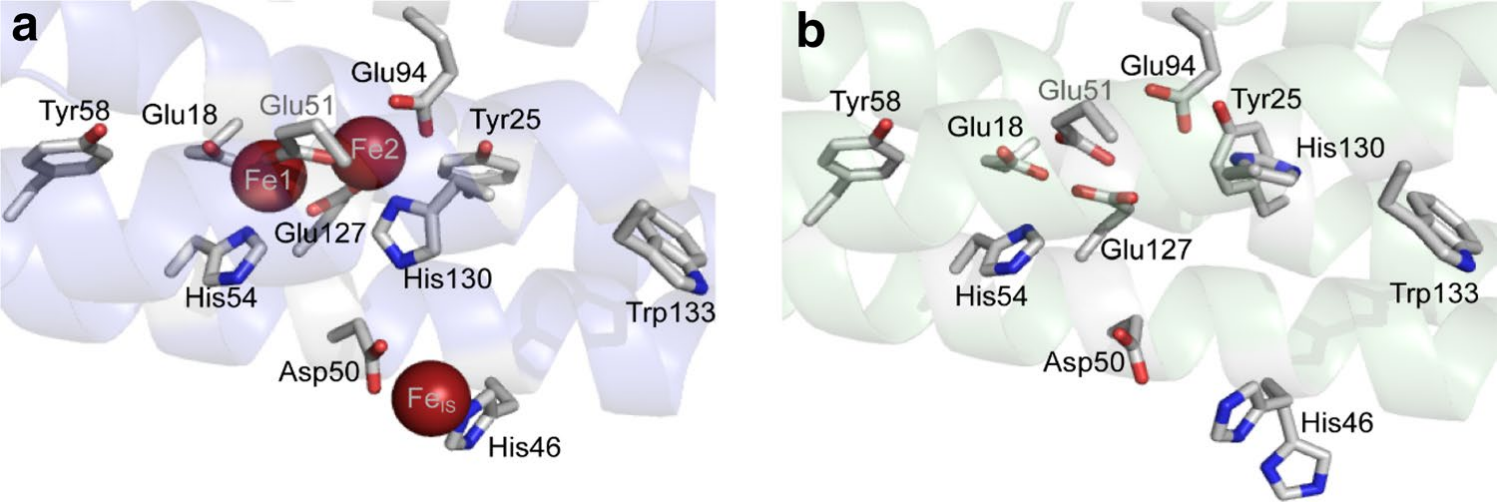
The ferroxidase center of EcBFR. **a** The diiron ferroxidase center of BFR is shown with coordinating residues (Glu18 and His54 are terminal ligands to Fe1; Glu94 and His130 are terminal ligands to Fe2; Glu51 and Glu127 bridge Fe1 and Fe2), along with the inner surface iron site (Fe_IS_) with coordinating residues (His46 and Asp50), and closely lying aromatic residues (Tyr25, Tyr58 and Trp133). **b** The apo-form of the ferroxidase center of BFR showing that residues that act as ligands to the irons are located in similar positions, with the exception of His130, which, in this apo-structure, has swung away from the iron binding sites such that the center adopts an open conformation. **a** and **b** generated using PyMol with PDB files 3E1M and 3E1L, respectively

The intra-subunit ferroxidase center of *E. coli* BFR in an Fe^3+^-bound form has Fe1 and Fe2 both ligated by terminal glutamate and histidine residues, and by two bridging glutamate residues (Fig. 3). The inter-iron distance is 3.6 Å in the *E. coli* BFR structure [23], similar to that reported for the di-Fe^3+^ forms of the center in *D. desulfuricans* (3.7 Å) and *A. vinelandii* (3.5 Å) BFRs [15, 72]. In each case, there is additional bridging electron density that is consistent with an oxygen-containing bridging species. In the Fe^2+^-bound state there is an increase in the Fe1 to Fe2 distance towards ~4 Å with no additional bridging electron density between the irons [15, 23, 72]. In the absence of iron the ligands that form the ferroxidase center have the same configuration as for the iron-containing center in most X-ray structures. However, in two structures, those for apo-BFR in the presence of phosphate and the D132F variant, the side chain of His130 is oriented away from the empty iron-binding site similarly to the equivalent residue of *P. aeruginosa* BFR B (see below). Thus, as with *P. aeruginosa* BFR B, His130 appears to be conformationally flexible. An additional Fe^2+^-binding site has been observed in EcBFR with the Fe^2+^ ion coordinated by Asp50, His46 and three water molecules [23]. This site is located on the inner surface of each subunit, facing the cavity. The distance between this inner surface site and the nearest ferroxidase center iron is 9.2 Å (Fig. 3). Fe^3+^ has not been detected in X-ray structures at this additional site, here called the IS site (after inner surface). As described below, the IS site is important for transmitting electrons from Fe^2+^ ions in the cavity to the ferroxidase center, and perhaps also iron to the growing mineral.

**Fig. 4:**
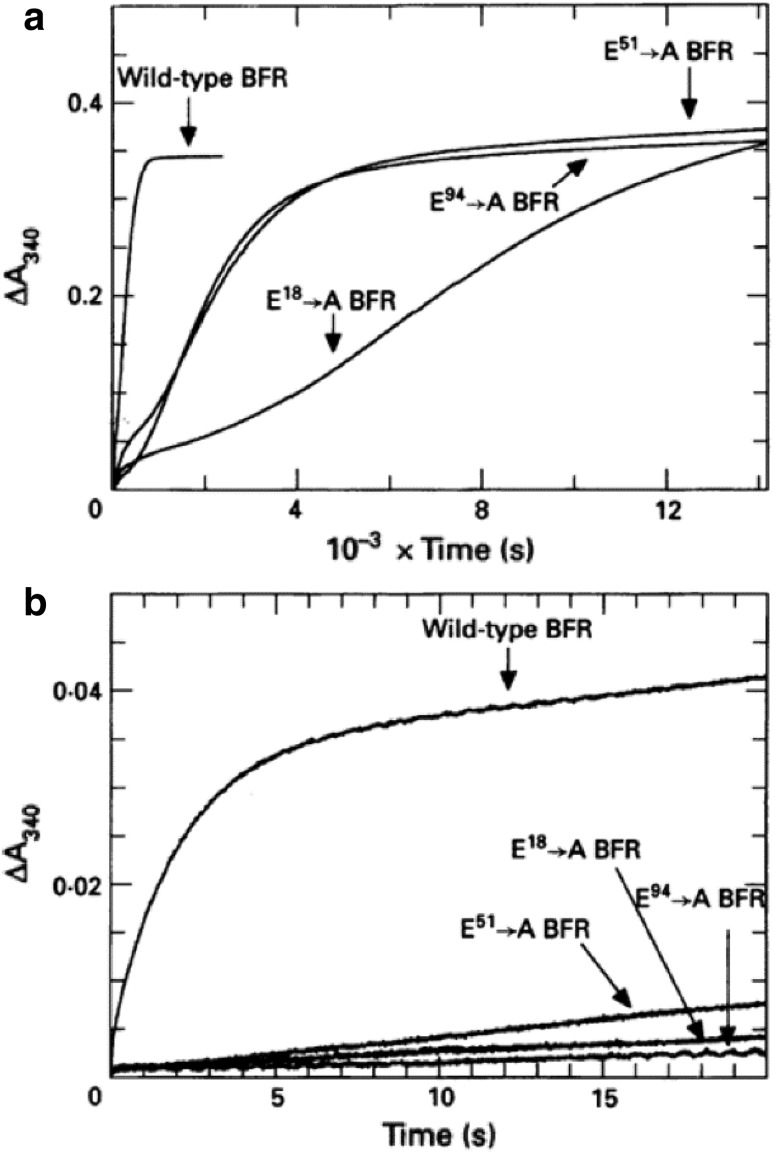
Kinetic traces for Fe oxidation in EcBFR. **a** Absorption change at 340 nm measured as a function of time after the addition of 400 Fe^2+^ ions per apo-protein molecule to samples of wild-type, E18A, E51A and E94A BFR, as indicated. For the wild-type protein this profile yields the phase 3 rate. **b** Absorption change at 340 nm followed by spectrophotometry over the first 20 s following the addition of 400 Fe^2+^ ions per apo-protein molecule. For the wild-type protein this profile yields the phase 2 rate. Proteins were in 100 mM MES buffer, pH 6.5 at a final concentration of 0.5 µM. Temperature was 30 °C, pathlength 1 cm. Reproduced with permission from Le Brun et al. [22]

Early studies of the aerobic addition of Fe^2+^ to *E. coli* apo-BFR identified three kinetic phases [73], the first of which, phase 1, is the binding of Fe^2+^ ions to BFR and the other two oxidation of Fe^2+^ ions catalyzed by BFR. The fast oxidation step, phase 2, saturates at two Fe^2+^ ions per subunit, and was later shown through mutagenesis studies to be oxidation of Fe^2+^ ions at the ferroxidase centers [22], which occurs before any iron is deposited in the central cavity. The slower oxidation step, phase 3, is associated with deposition of Fe^3+^ ions in the central cavity. Examples of the kinetic traces observed are given in Fig. 4. These show the time dependence of the increase in absorbance at 340 nm associated with oxidation of Fe^2+^ to Fe^3+^ for wild-type BFR and three ferroxidase center variants. The lower traces show the rapid phase 2 reaction of wild-type BFR, which is absent from the ferroxidase center variants, and the upper trace the slower phase 3 reactions. Two points are notable from these data. First, for rapid oxidation of Fe^2+^ ions intact ferroxidase centers are essential, and second, that intact ferroxidase centers are not essential for complete oxidation of the added Fe^2+^ to Fe^3+^. This latter point is shown by the similar overall absorbance changes for wild-type BFR and the three variants. This is one of the earliest indications that while rapid oxidation of Fe^2+^ ions by BFR requires functioning ferroxidase centers, at least one other Fe^2+^ ion oxidation pathway exists in BFR that does not need the ferroxidase center. This may well involve the surface of a growing Fe^3+^ core in the cavity acting to catalyze Fe^2+^ oxidation, a mechanism widely discussed in the ferritin literature and relevant to the mechanism of core formation in human ferritin put forward by Pan et al. [36] and discussed above, but not one considered further here (for example, [50]). An important observation in these early kinetic studies was that the phase 2 reaction was only observed once per apo-BFR sample, suggesting that some or all of the Fe^3+^ product remained in the ferroxidase center preventing further Fe^2+^ ions from binding and being oxidized there [22]. EPR studies supported this by showing that a significant fraction of the added Fe^2+^ ions was not visible as mononuclear Fe^3+^ following oxidation. Several models were put forward to explain these data with the simplest being that the Fe^2+^ dimers occupied the ferroxidase centers and became oxidized with some of the product Fe^3+^ dimers breaking up. Later EPR studies [74] quantified the amount of monomeric Fe^3+^ ions following the oxidation of the first 48 Fe^2+^ ions per apoBFR and found that only 3 % of the iron in BFR was monomeric, leading to the conclusion that the monomeric Fe^3+^ complex is not a major product of the ferroxidase reaction. Structural studies were entirely in agreement with this in that iron-soak experiments with EcBFR crystals revealed full occupancy of the ferroxidase sites in both Fe^2+^ and Fe^3+^ states [23]. Furthermore, recent circular dichroism (CD) and magnetic CD data confirmed that upon O_2_ redox cycling the diiron ferroxidase center remains intact while the IS site is vacated [31].

Though the majority of mechanistic studies of EcBFR, indeed, all ferritins, have concentrated on the Fe^2+^ substrate the nature of the oxidant is also important. Despite our comments above about the laying down of Fe^3+^ cores in some bacterial cells grown anaerobically, in vitro studies have largely concentrated on the use of O_2_ as an oxidant, and occasionally H_2_O_2_ [75]. Oximetry measurements [74] with EcBFR have shown that the ratio of Fe^2+^ oxidised to O_2_ reduced is 4, indicating that all the electrons for the reduction of the O_2_ come from Fe^2+^ oxidation. H_2_O_2_ is a more efficient oxidant of the EcBFR di-Fe^2+^ ferroxidase center but this does not affect the Fe^2+^:O_2_ stoichiometry in competition experiments with O_2_ [75].

**Fig. 5:**
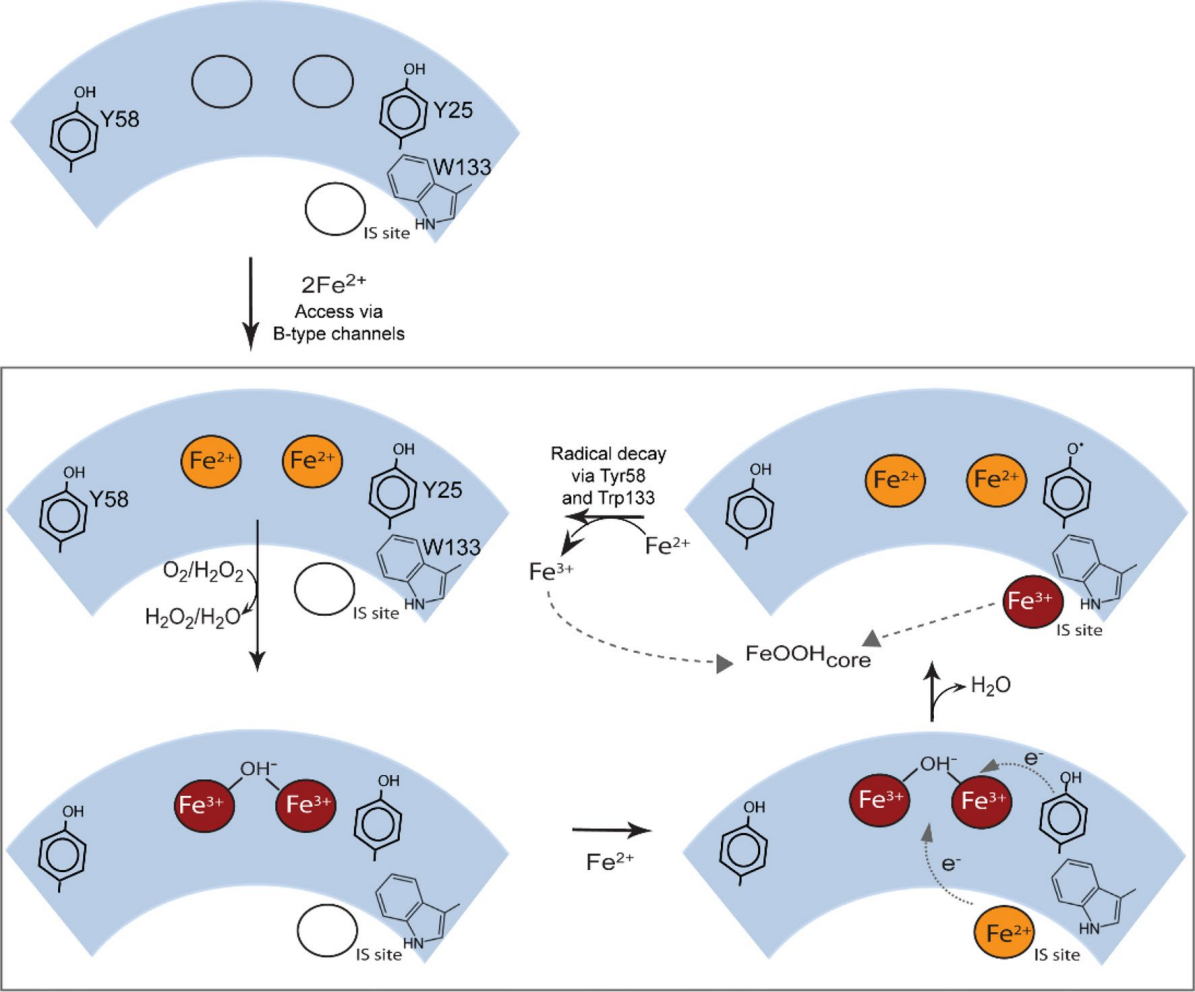
Summary of the proposed BFR mineralization mechanism. Two Fe^2+^ ions access the ferroxidase site via B-type channels [16] and undergo oxidation to the bridged di-ferric form (with either O_2_ or H_2_O_2_ as oxidant [75]. The oxidized di-Fe^3+^ form of the site is stable [23]. Additional Fe^2+^ ions binds at the inner surface site (IS site) and undergoes oxidation to Fe^3+^. A second electron is derived from the oxidation of the nearby Tyr25 side chain, generating a radical and regenerating the di-Fe^2+^ form of the ferroxidase site. The radical decays indirectly through the oxidation of a second Fe^2+^ ion (at an unknown location), and the oxidized iron at the inner surface site nucleates or is incorporated into the growing mineral core. The di-Fe^2+^ ferroxidase site undergoes oxidation again via reaction with O_2_ or H_2_O_2_. At this point, the catalytic site has returned to its resting state, ready to react again when Fe^2+^ ions are present. Hydrolysis of the accumulating hydrated Fe^3+^ ions in the cavity leads to mineral formation. Adapted from Bradley et al. [76]

As noted above, ferroxidase activity in EcBFR is essential throughout rapid core formation and this, together with the failure to detect a second round of phase 2 activity after the addition of 48 Fe^2+^ ions to apo-BFR, and the stability of the oxidized form of the dinuclear center evident from kinetic, crystallographic and EPR studies [22, 23, 74], and now CD and MCD data [31], led to a proposed mechanistic model (Fig. 5) for mineralization in which the ferroxidase site functions as a true catalytic center, continually cycling between its oxidized (bridged di-Fe^3+^) and reduced (di-Fe^2+^) forms. In this model [76], the catalytic cycling of the ferroxidase center is driven by the oxidation of Fe^2+^ ions in the central cavity, with the electrons resulting from this passing to the oxidized ferroxidase center to effect its reduction back to the di-Fe^2+^ form, which is then primed to react again with O_2_ (or H_2_O_2_). This model requires the existence of an electron transfer route from the cavity to the ferroxidase center, and at least some elements of this have now been established (Fig. 5). Disruption of the IS site described above by singly replacing His46 and Asp50 with alanines did not affect the rapid oxidation of Fe^2+^ ions at the ferroxidase site, phase 2, but did severely inhibit subsequent mineralization, phase 3, suggesting that the IS site forms part of an electron transfer pathway between Fe^2+^ ions in the cavity and the ferroxidase center, with Fe_IS_ becoming incorporated in the growing core. More recently, we found that mineralization in EcBFR depends on three aromatic residues near to the ferroxidase center, Tyr25, Tyr58 and Trp133, and that a transient radical detectable by EPR is formed on Tyr25. The key kinetic observations were that the single site-directed variants, in which each residue was substituted with phenylalanine, had phase 2 activities similar to that of wild-type BFR but considerably impaired phase 3 activities upon the addition of 400 Fe^2+^ ions per 24mer, with mineralization rates of 15 % (Y25F), 30 % (Y58F), and 25 % (W133F) relative to wild type BFR [76]. The observations of the importance of the IS site and the detection of a transient radical species associated with Tyr25 provide important evidence about the origin of the two electrons required to reduce the oxidized ferroxidase site. We proposed [76] that one electron comes from the oxidation of the inner surface site Fe^2+^, while the other is from Tyr25, generating the radical, which is then quenched by an electron from a second Fe^2+^ ion that is probably located in the cavity. The net effect of this is that two Fe^2+^ ions in the cavity are oxidized with the delivery of two electrons to the di-Fe^3+^ ferroxidase center (Fig. 5). This mechanism enables the near simultaneous arrival of the two electrons at the oxidized ferroxidase site, which minimises the possibility of single electron reduction of O_2_ or H_2_O_2_, and the accidental release of toxic reactive oxygen species, in keeping with a role for EcBFR in controlling oxidative stress.

The issue of how Fe^2+^ ions access the empty BFR protein shell has been addressed with site-directed mutagenesis [16]. Blocking the B-channels by mutating Asp132 to Phe (Fig. 2) has been most revealing. While the structure of the D132F variant was unaffected beyond the substituted side chain, the rates of both the phase 2 and phase 3 reactions were significantly reduced compared to the wild-type rates, indicating that the B-channels are a major route for the entry of iron to the ferroxidase center and the central cavity of EcBFR.

The assay conditions used to explore the aerobic core formation in the previous paragraphs generate a more crystalline, and relatively phosphate-free core, than is observed for native EcBFR. Aitken-Rogers et al. [77] explored this by adding phosphate to the solutions for in vitro core formation under similar conditions to those employed for the mechanistic studies leading to the proposed mechanism set out in Fig. 5. The key findings from this [77] study were: (i) The iron core had a composition and EPR properties characteristic of native cores, unlike the core generated under solution conditions without added phosphate; (ii) Phase 2 Fe^2+^ oxidation—i.e. occupation of the ferroxidase center by two Fe^3+^ ions—occurred similarly in the presence or absence of added phosphate; (iii) Phase 3 oxidation rates were significantly enhanced in the presence of phosphate. In terms of the mechanism set out in Fig. 5, this suggest that the rate–limiting steps of the core formation reaction lies with events at the core rather than with reduction of O_2_ at the ferroxidase center. EPR studies [78] suggested that phosphate plays a role in shepherding iron into the central cavity. Therefore, in terms of the key mechanistic steps involved in Fe^2+^ oxidation and O_2_ reduction, the low-phosphate in vitro conditions employed by Bradley et al. [76] probably mimic the key stages in the physiological build-up of an iron core in terms of the intermediates involved.

## Mechanistic studies of *Pseudomonas aeruginosa* Bacterioferritin B (PaBFR)

Rivera and his colleagues have shown that *P. aeruginosa* BFR B lays down an iron core differently to EcBFR despite the marked structural similarity between the two (70 % identity), which extends to the same residues forming the ferroxidase center in both [27]. However, unlike with EcBFR, the ferroxidase center of PaBFR is unstable and its occupation by iron has only been detected in the X-ray structure of crystals soaked in a solution containing Fe^2+^. In this di- Fe^2+^ structure, the Fe–Fe distance is ~4 Å with no bridging electron density between the irons, as seen for EcBFR (Fig. 3). A striking finding from the X-ray structures is that the orientation of His130 relative to the other ferroxidase center ligands is variable. In the iron loaded center the His130 side chain is bound to an iron ion but in the metal-free site it is rotated away from the ferroxidase center, similarly to His130 of D132F EcBFR [16]. Rivera and his colleagues [27] call the two conformations of His130 in PaBFR the “gate open” and “gate closed” conformations, with the latter being the conformation bound to an iron ion in the filled ferroxidase center. The switch between the two conformations is an important step in their mechanism of core formation in this ferritin.

There are substantial difference between *E. coli* and *P. aeruginosa* BFRs in the kinetics of their aerobic oxidative accumulation of Fe^2+^ ions. Though PaBFR, like EcBFR, has two phases at low ratios of Fe^2+^:BFR (≤100) the slower phase, which is due to mineralization in the central cavity, becomes progressively faster as the ratio is increased and eventually obscures the initial fast phase [27]. However, at low ratios the fast phase does not saturate and the transition between the fast and slow phases is accompanied by a small decrease in the absorbance change associated with oxidation of Fe^2+^ ions. Weeratunga et al. [27] conclude from these observations that the fast phase corresponds to the oxidation of the di-Fe^2+^ ferroxidase center with the decrease in absorbance resulting from migration of the Fe^3+^ ions produced into the central cavity. The switch between the “gate open” and “gate closed” conformations of His130 is important for this migration because when the gate is open there is a direct path between the ferroxidase center and the internal cavity. We note that the same flexibility of His130 in EcBFR is also considered functionally important but for entry of Fe^2+^ ions into the ferroxidase center rather than exit of Fe^3+^ ions [16].

## Mechanistic studies of *Pseudo*-*nitzschia multiseries* ferritin (PmFTN)

**Fig. 6:**
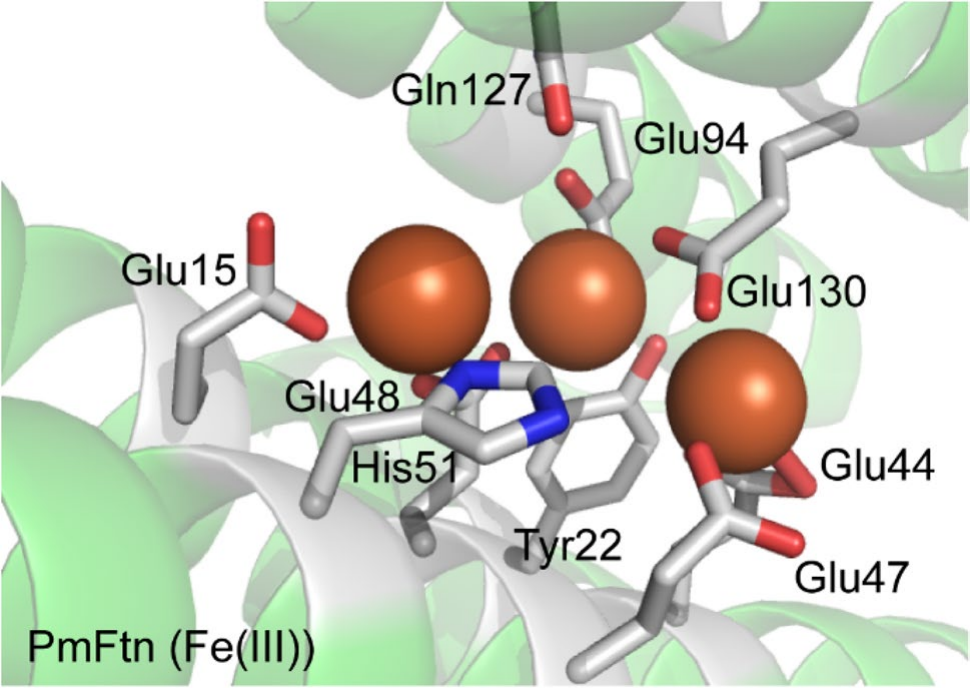
The Ftn-type ferroxidase center and site C of *P. multiseries* Ftn. Structure of the eukaryotic Ftn-type ferroxidase center and site C of *P. multiseries* Ftn with Fe^3+^ ions bound generated using PyMol from PDB 4IWK [79]

*Pseudo*-*nitzschia multiseries* is a marine pennate diatom that plays a major role in global primary production and carbon sequestration in the deep ocean. Its ferritin is important for sustaining its growth in iron-limited environments [79] and perhaps for this reason it appears that PmFTN is optimized for initial Fe^2+^ oxidation and not for mineralization of iron. Thus it may have a role in buffering iron availability and facilitating iron-sparing, the response of a cell to low levels of iron, rather than simply long-term iron storage. The X-ray structure of PmFTN shows it has a ferroxidase center and an additional iron-binding site, site C, nearby (Fig. 6). This arrangement is typical for bacterial FTNs [9] and PmFTN is the first observation of it in a eukaryotic ferritin. The PmFTN site C is located closer to the ferroxidase center than is seen with the site C of prokaryotic FTNs but it shares two common ligands with them (Glu47 and Glu130). Glu130 of PmFTN connects a ferroxidase center iron at site B with C, and Glu44 is an iron ligand both at site C and on the inner surface of the protein shell.

X-ray studies of crystals soaked in iron-containing solutions under varying conditions along with kinetic studies showed that Fe^2+^ ions bind stepwise in a dioxygen-dependent manner, with the binding of the second iron ion the trigger for oxidation to occur. Glu130 and Glu44 were proposed to shuttle metal ions between sites and this proposal led Pfaffen et al. [79] to carry out a mutagenesis study to investigate further the roles of these glutamates. As with the BFRs described above, the kinetics of the aerobic oxidation of Fe^2+^ ion catalyzed by PmFTN was central to the investigation. Like EcBFR, apo-PmFTN has a detectable phase 2 reaction associated with the oxidation of Fe^2+^ to Fe^3+^ at the ferroxidase center and a slower phase 3 reaction, which is formation of the core mineral [79, 80]. The rate of the phase 2 reaction is amongst the highest reported for any ferritin. With the addition of two Fe^2+^ ions per subunit, the *t*_½_ for the phase 2 reaction was <50 ms, which is an order of magnitude faster than those measured for *E. coli* FtnA and human H ferritin under comparable conditions. The rate of the phase 2 reaction is so fast that Pfaffen et al. [79] suggest that Fe^2+^ binding, rather than Fe^2+^ oxidation, is the rate-determining step of the reaction.

**Fig. 7:**
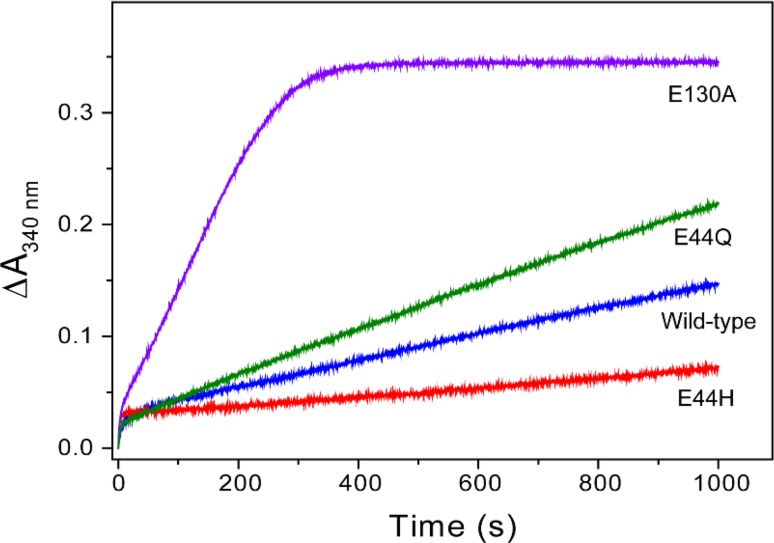
Stopped -flow spectroscopy of iron mineralization in wild type and variant PmFTN Absorbance changes at 340 nm showing Fe^2+^ oxidation following addition of 400 Fe^2+^/PmFTN to wild type and variant PmFTN (0.5 μM) in 0.1 MES pH 6.5 at 25 °C. The profile of the plots from ~50 s onwards is determined by the rate of the phase 3 reactions. With the E130A variant this is complete in about 300 s but with the wild-type protein it is only about 40 % done in 1000 s. Note that in EcBFR under similar conditions the phase 3 reaction with 400 Fe^2+^/EcBFR is complete in about 1000 s (Fig. 4a). Reproduced with permission from Pfaffen et al. [80]

The ability of the variants to mineralize iron was investigated following addition of 400 Fe^2+^ ions per apo-protein (Fig. 7). The initial rates of the phase 3 reaction were similar for E44Q and wild-type PmFTN, whereas mineralization in E44H PmFTN was significantly slower. The remarkable finding, however, was that mineralization in the E130A variant occurred ~10-fold faster than in wild-type PmFTN. In all these variants the rate of the phase 2 reaction was not much changed from the wild-type protein. These data indicate that PmFTN has evolved to oxidise Fe^2+^ ions at the ferroxidase center extremely rapidly, and to hold it there in a partially stable form, rather than rapidly transfer it to the central cavity for mineralization [80]. These observations provide clear experimental data in support of the proposal that the function of site C, at least in some ferritins, is to regulate the flux of Fe^3+^ ions through the ferroxidase site, as proposed by Treffry et al. [81]. The benefit of this in the case of the diatom ferritin may be to increase the availability of iron taken up by ferritin for rapid redeployment of a scarce resource. In such an iron-buffering role, core formation in the internal cavity could be less important.

## Furnishing other proteins with iron from ferritins

At various points in this article we have referred to ferritins as iron donors to other proteins and in this section we want to briefly consider this aspect. For ferritin to release iron without damaging itself, the core Fe^3+^ needs to be reduced. The product Fe^2+^ ions then have to traverse the protein coat and be released to acceptor molecules outside the ferritin. Thus, as with the aerobic uptake of Fe^2+^ ions into ferritins, the release of Fe^2+^ ions from ferritins is linked to redox reactions. An important consideration in redox reactions is the relative redox potentials of the electron donor and acceptor species. Watt and his colleagues have reported these for the cores of *A. vinelandii* BFR and horse spleen ferritin to be −420 ± 20 mV at pH 7–9 and ~−190 mV at pH 7, respectively [44, 82], suggesting that if we consider just the reduction of the core Fe^3+^ its physiological reductants would need to have low redox potentials. However, the reduction of the core Fe^3+^ and release of product Fe^2+^ ions to acceptor molecules are coupled events and the affinity of the Fe^2+^ ions for the acceptor molecules is important. This can be seen by considering the basic scheme of the commonly employed in vitro reductive iron release assays (Eqs. 1, 2) [83–85]:

1 $$ {\text{Core }}{-}{\text{ Fe}}^{ 3+ }_{\text{n}} + {\text{ e}}^{ - } \to {\text{Core }}{-}{\text{ Fe}}^{ 3+ }_{{({\text{n}} - 1)}} + {\text{ Fe}}^{ 2+ } $$

2 $$ {\text{Fe}}^{ 2+ } + {\text{ xL}} \to {\text{Fe}}^{ 2+ } \left( {\text{L}} \right)_{x} $$

The electron is usually provided by a small molecule reactant such as dithionite, flavin or a quinol, and the iron acceptor molecule (L) is generally ferrozine or bipyridyl, which yields a colored product whose formation can be monitored spectrophotometrically. Reaction (1) is normally slow and reaction (2) is much faster so that the overall rate of formation of Fe^2+^(L)_*x*_ corresponds to the rate of reaction (1) with the equilibrium constant for the overall scheme dominated by the equilibrium constant for reaction (2). The significance of the iron acceptor molecule in this scheme is thus considerable which means that for physiologically relevant iron release studies, ideally the physiological acceptor molecules will be employed. This is in contrast to the aerobic iron uptake assays we have already considered, and is a consequence of the thermodynamic driving force for uptake generally being the downhill formation of Fe^3+^ species within ferritin whilst the driving force for iron release is the formation of the Fe^2+^ acceptor molecule complex. As with aerobic uptake of Fe^2+^ ions, in which long-range electron transfer through the protein is now recognized to be an important feature (e.g. Fig. 5), it appears that electron transfer through the ferritin protein is important in reductive release of Fe^2+^ ions, at least in the small molecule studies using the assays of reactions (1) and (2). This is shown by the dependence of the overall rate of formation of Fe^2+^(L)_*x*_ on the redox potential of the electron donor [84, 85].

Iron donor molecules to ferritins and iron acceptor molecules from ferritins could be examples of metallochaperones, iron binding proteins whose function is the intracellular trafficking of iron between molecules. First identified for Cu^2+^ ions, metallochaperones are thought to be important for other redox-active metal ions, including iron [29, 86]. The clearest example of iron chaperones that work with animal ferritins has come from studies by Philpott and her colleagues with the Poly(rC)-binding proteins PCBP1 and PCBP2 [29, 87]. PCBP1 was originally identified as an RNA binding protein but in 2008 Shi et al. [30] reported that it delivered iron to ferritin in human cells, and later studies [87] indicated that PCBP1 and PCBP2 delivered iron to many iron-requiring enzymes. Where PCBP1 and PCBP2 obtain the iron they pass on to ferritin and other enzymes has not been firmly established but at least some of it comes from a pool of labile iron present in the cell. Whether PCBP1 and PCBP2 are capable of acting as iron acceptors from ferritin has not been reported but if they are not the iron acceptor chaperones then presumably there are other iron chaperones waiting to be discovered!

BFR was the first ferritin for which a partner protein was identified, the so-called Bacterioferritin-associated ferredoxin, Bfd, which contains a single 2Fe/2S cluster [56, 88, 89]. A significant breakthrough in characterizing this came with the X-ray structure of *P. aeruginosa* Bfd bound to its BFR [90, 91]. The 24mer BFR bound 12 Bfd molecules at independent and identical binding sites that placed each 2Fe/2S cluster relatively close to one of the 12 heme groups of the BFR. Parallel mechanistic studies [27, 90, 91] showed that the presence of the bound Bfd helped mobilize iron from the core of BFR by feeding electrons to it via the heme groups. Since the gene for Bfd is widespread in bacteria it is likely that reaction described by Yao et al. [90] is common. Above we have described the driving force for iron release to be the formation of the Fe^2+^ acceptor molecule complex, and with *A. vinelandii* BFR supporting evidence for this is that protein with a core of 1000–2000 Fe^2+^ ions can be passed through gel filtration columns and studied spectroscopically without significant loss of iron provided a chelator is not present [82]. This leads on to the intriguing thought that if BFR also has an iron acceptor protein within the cell, reductive iron release will involve not only Bfd but also another protein so a complex of three kinds of protein may be involved. However, a prokaryotic iron chaperone has not been reported.

Up to now in this section we have described iron release from ferritins without damage to the protein coat but it has been known for many years that ferritin within animal cells can become damaged and get converted to an insoluble iron-rich aggregate commonly called hemosiderin [92, 93]. How hemosiderin is formed is not understood but it is known it involves ferritin being transported to lysosomes and degraded. The recent striking discovery of ferritinophagy [94–96], an intracellular degradative process whereby iron is released from ferritin in mammalian cells, may be related to the formation of hemosiderin. Ferritinophagy involves ferritin interacting with the nuclear receptor coactivator 4 protein, NCOA4, which directs the ferritin to lysosomes were it is degraded in an as yet undefined manner to release iron for use by the cell [96]. Whether degradative pathway are the only ones to exist in animals for release of iron from ferritins has not been established but if a reductive pathway exists that leaves the ferritin undamaged and able to function normally then the relative fluxes of iron through the different pathways will be important to determine.

## Concluding remarks

In this review we have sought to discuss iron uptake into ferritins in a manner that illustrates the mechanistic diversity that exists in this superfamily of proteins for what at first sight appears to be a common chemical event, the laying down of a polynuclear iron species in the central cavity of a common protein structure. The principle differences amongst our three ferritins we want to highlight are:

1. The ferroxidase centre of EcBFR is stable once formed and functions as a catalytic site for O_2_ reduction;
2. The apparently identical ferroxidase site of PaBFR is not stable and functions as a catalytic site for O_2_ reduction and a transit site for passage of Fe^3+^ ions into the central cavity;
3. PmFTN oxidises Fe^2+^ ions at its ferroxidase center extremely rapidly and only slowly builds up a core consistent with a major role in iron buffering rather than long-term iron storage.

Thus, just considering mechanistic details of three ferritins shows that even when there is a considerable degree of structural similarity, which can even extend to the identity and conformation of inner-sphere ligands coordinating iron at a key functional site, mechanistic differences can be considerable for aerobic uptake of iron ions. This leads on to the question of what the origin of the mechanistic differences can be, since it clearly can’t be simply the coordination sphere of the bound iron. Thus we have to consider outer-sphere effects. For example, do the coordinating ligands of different ferritins have similar hydrogen bonding interactions? As far as *E. coli* and *P. aeruginosa* BFRs are concerned the answer is yes [23, 27], so this cannot account for the differing properties of their ferroxidase centers. So perhaps long-range effects resulting from electrostatic interactions and/or differing molecular dynamics modulate their chemical properties? However, to date a full consideration of these issues has not been reported, doubtless a reflection of the difficulty in actually carrying out these kinds of studies. What has been established thus far is that the electrostatic profiles of channels through the protein shells of different ferritins differ considerably [4, 16, 97, 98], and the dynamical properties of the channels of PaBFR [99] and the dynamical and electrostatic properties of frog ferritin [8, 65, 100] influence the manner in which these proteins accumulate iron. Similar studies with other ferritins are required to probe these aspects further.

We have suggested that the mechanistic differences within the ferritin superfamily reflect differing evolutionary pressures on amino acid sequences, and that these differing pressures are a consequence of different primary functions for different ferritins. Evidence to support this notion is not strong but it is clear that different ferritins do have different primary functions—not all have as their chief goal iron storage as part of a general house-keeping function—and thus evolutionary pressures on them are expected to be different.

## Abbreviations

BFR: Bacterioferitin
EM: Electron microscopy
EPR: Electron paramagnetic resonance spectroscopy
FTN: Prokaryotic ferritin

## References

[1] Laufberger V (1937). Bull Soc Chim biol.

[2] Lewis CT, Short C (1879) A latin dictionary. Clarendon Press, Oxford. http://www.perseus.tufts.edu. Accessed 3 Jan 2016

[3] Ford GC, Harrison PM, Rice DW, Smith JMA, Treffry A, White JL, Yariv J (1984). Philos Trans R Soc Lond Ser.

[4] Harrison PM, Arosio P (1996). Biochim Biophys Acta.

[5] Lewin A, Moore GR, Le Brun NE (2005) Dalton Trans, pp 3597–3610 10.1039/b506071k 16258608

[6] Theil EC, Matzapetakis M, Liu XF (2006). J Biol Inorg Chem.

[7] Andrews SC (2010). Biochim Biophys Acta.

[8] Theil EC (2013). Inorg Chem.

[9] Bradley JM, Moore GR, Le Brun NE (2014). J Biol Inorg Chem.

[10] Arosio P, Carmona F, Gozzelino R, Maccarinelli F, Poli M (2015). Biochem J.

[11] Fischbach FA, Anderegg JW (1965). J Mol Biol.

[12] Granick S, Michaelis L (1943). J Biol Chem.

[13] Stiefel EI, Watt GD (1979). Nature.

[14] Andrews SC, Le Brun NE, Barynin V, Thomson AJ, Moore GR, Guest JR, Harrison PM (1995). J Biol Chem.

[15] Macedo S, Romao CV, Mitchell E, Matias PM, Liu MY, Xavier AV, LeGall J, Teixeira M, Lindley P, Carrondo MA (2003). Nat Struct Biol.

[16] Wong SG, Grigg JC, Le Brun NE, Moore GR, Murphy ME, Mauk AG (2015). J Biol Chem.

[17] Lawson DM, Treffry A, Artymiuk PJ, Harrison PM, Yewdall SJ, Luzzago A, Cesareni G, Levi S, Arosio P (1989). FEBS Lett.

[18] Lawson DM, Artymiuk PJ, Yewdall SJ, Smith JMA, Livingston JC, Treffry A, Luzzago A, Levi S, Arosio P, Cesareni G, Thomas CD, Shaw WV, Harrison PM (1991). Nature.

[19] Ha Y, Shi D, Small GW, Theil EC, Allewell NM (1999). J Biol Inorg Chem.

[20] Tosha T, Ng H-L, Bhattasali O, Alber T, Theil EC (2010). J Am Chem Soc.

[21] Toussaint L, Bertrand L, Hue L, Crichton RR, Declercq JP (2007). J Mol Biol.

[22] Le Brun NE, Andrews SC, Guest JR, Harrison PM, Moore GR, Thomson AJ (1995). Biochem J.

[23] Crow A, Lawson TL, Lewin A, Moore GR, Le Brun NE (2009). J Am Chem Soc.

[24] Hempstead PD, Hudson AJ, Artymiuk PJ, Andrews SC, Banfield MJ, Guest JR, Harrison PM (1994). FEBS Lett.

[25] Stillman TJ, Hempstead PD, Artymiuk PJ, Andrews SC, Hudson AJ, Treffry A, Guest JR, Harrison PM (2001). J Mol Biol.

[26] Honarmand Ebrahimi K, Hagedoorn PL, Hagen WR (2015) Chem Rev 115:295–326 10.1021/cr5004908 25418839

[27] Weeratunga SK, Lovell S, Yao H, Battaile KP, Fischer CJ, Gee CE, Rivera M (2010). Biochemistry.

[28] Kwak Y, Schwartz JK, Huang VW, Boice E, Kurtz DM Jr, Solomon EI (2015) Biochemistry 54:7010–7018 10.1021/acs.biochem.5b01033 26551523

[29] Philpott CC (2012). J Biol Chem.

[30] Shi H, Bencze KZ, Stemmler TL, Philpott CC (2008). Science.

[31] Vasil ML, Ochsner UA (1999). Mol Microbiol.

[32] Bereswill S, Greiner S, van Vliet AH, Waidner B, Fassbinder F, Schiltz E, Kusters JG, Kist M (2000). J Bacteriol.

[33] Yariv J (1983). Biochem J.

[34] Kimura M (1983) The neutral theory of molecular evolution. Cambridge University Press, Cambridge

[35] St Pierre TG, Tran KC, Webb J, Macey DJ, Heywood BR, Sparks NH, Wade VJ, Mann S, Pootrakul P (1991) Biol Met 4:162–165 10.1007/BF01141308 1931435

[36] Pan YH, Sader K, Powell JJ, Bleloch A, Gass M, Trinick J, Warley A, Li A, Brydson R, Brown A (2009). J Struct Biol.

[37] Granick S (1942). J Biol Chem.

[38] Fischbach FA, Harrison PM, Hoy TG (1969). J Mol Biol.

[39] Mann S, Bannister JV, Williams RJP (1986). J Mol Biol.

[40] St Pierre TG, Kim KS, Webb J, Mann S, Dickson DPE (1990) Inorg Chem 29:1870–1874

[41] Wade VJ, Treffry A, Laulhère JP, Bauminger ER, Cleton MI, Mann S, Briat JF, Harrison PM (1993) Biochim Biophys Acta 1161:91–96 10.1016/0167-4838(93)90201-2 8422424

[42] Barceló F, Otero Areán C, Moore GR (1995). Biometals.

[43] Doig P, Austin JW, Trust TJ (1993). J Bacteriol.

[44] Watt GD, Frankel RB, Papaefthymiou GC, Spartalian K, Stiefel EI (1986). Biochemistry.

[45] Ringeling PL, Davy SL, Monkara FA, Hunt C, Dickson DPE, McEwan AG, Moore GR (1994). Eur J Biochem.

[46] Moore GR, Mann S, Bannister JV (1986). J Inorg Biochem.

[47] St Pierre TG, Bell SH, Dickson DPE, Mann S, Webb J, Moore GR, Williams RJP (1986) Biochim Biophys Acta 870:127–134 10.1016/0167-4838(86)90015-4 3081032

[48] Cheesman MR, Kadir FHA, Al-Basseet J, al-Massad F, Farrar J, Greenwood C, Thomson AJ, Moore GR (1992). Biochem J.

[49] Theil EC (1973). J Biol Chem.

[50] Chasteen ND, Harrison PM (1999). J Struct Biol.

[51] Moore GR, Kadir FH, al-Massad FK, Le Brun NE, Thomson AJ, Greenwood C, Keen JN, Findlay JB (1994) Biochem J 304:493–497 10.1042/bj3040493 PMC1137519 7998985

[52] al-Massad FK, Kadir FH, Moore GR (1992) Biochem J 283:177–180 10.1042/bj2830177 PMC1131011 1567365

[53] Kadir FH, Read NM, Dickson DP, Greenwood C, Thompson A, Moore GR (1991). J Inorg Biochem.

[54] Reid NM, Dickson DP, Greenwood C, Thompson A, Kadir FH, Moore GR (1990). Biochem J.

[55] Bauminger ER, Cohen SG, Dickson DPE, Levy A, Ofer S, Yariv J (1980). Biochim Biophys Acta.

[56] Abdul-Tehrani H, Hudson AJ, Chang YS, Timms AR, Hawkins C, Williams JM, Harrison PM, Guest JR, Andrews SC (1999). J Bacteriol.

[57] Mann S, Williams JM, Treffry A, Harrison PM (1987). J Mol Biol.

[58] Rohrer JS, Islam QT, Watt GD, Sayers DE, Theil EC (1990). Biochemistry.

[59] Massover WH (1993). Micron.

[60] Papaefthymiou GC (2010). Biochim Biophys Acta.

[61] Wajnberg E, El-Jaick LJ, Linhares MP, Esquivel DM (2001). J Magn Reson.

[62] Le Brun NE, Moore GR, Thomson AJ (1995). Mol Phys.

[63] Harrison PM, Fischbach FA, Hoy TG, Haggis GH (1967). Nature.

[64] Sadeghi O, Zakharov LN, Nyman M (2015). Science.

[65] Behera RK, Theil EC (2014). Proc Natl Acad Sci.

[66] Theil EC (2011). Curr Opin Chem Biol.

[67] Wade VJ, Levi S, Arosio P, Treffry A, Harrison PM, Mann S (1991). J Mol Biol.

[68] Levi S, Santambrogio P, Cozzi A, Rovida E, Corsi B, Tamborini E, Spada S, Albertini A, Arosio P (1994). J Mol Biol.

[69] López-Castro JD, Delgado JJ, Perez-Omil JA, Gálvez N, Cuesta R, Watt RK, Domínguez-Vera JM (2012). Dalton Trans.

[70] Yao H, Jepkorir G, Lovell S, Nama PV, Weeratunga S, Battaile KP, Rivera M (2011). Biochemistry.

[71] Ma J-F, Ochsner UA, Klotz MG, Nanayakkara VK, Howell ML, Johnson Z, Posey JE, Vasil ML, Monaco JJ, Hasseett DJ (1999). J Bacteriol.

[72] Swartz L, Kuchinskas M, Li HY, Poulos TL, Lanzilotta WN (2006). Biochemistry.

[73] Le Brun NE, Wilson MT, Andrews SC, Guest JR, Harrison PM, Thomson AJ, Moore GR (1993) FEBS Lett 333:197–202 **(323:261–266)** 10.1016/0014-5793(93)80404-i 8224163

[74] Yang X, Le Brun NE, Thomson AJ, Moore CR, Chasteen ND (2000). Biochemistry.

[75] Bou-Abdallah F, Lewin AC, Le Brun NE, Moore GR, Chasteen ND (2002). J Biol Chem.

[76] Bradley JM, Svistunenko DA, Lawson TL, Hemmings AM, Moore GR, Le Brun NE (2015). Angew Chem Int Ed Engl.

[77] Aitken-Rogers H, Singleton C, Lewin A, Taylor-Gee A, Moore GR, Le Brun NE (2004). J Biol Inorg Chem.

[78] Le Brun NE, Cheesman MR, Thomson AJ, Moore GR, Andrews SC, Guest JR, Harrison PM (1993). FEBS Lett.

[79] Pfaffen S, Abdulqadir R, Le Brun NE, Murphy ME (2013). J Biol Chem.

[80] Pfaffen S, Bradley JM, Abdulqadir R, Firme MR, Moore GR, Le Brun NE, Murphy ME (2015) J Biol Chem 290:28416–28427 10.1074/jbc.M115.669713 PMC4653698 26396187

[81] Treffry A, Zhao Z, Quail MA, Guest JR, Harrison PM (1998). FEBS Lett.

[82] Watt GD, Frankel RB, Papaefthymiou GC (1985). Proc Natl Acad Sci.

[83] Jones T, Spencer R, Walsh C (1979). Biochemistry.

[84] Boyer RF, Clark HM, LaRoche AP (1988). J Inorg Biochem.

[85] Kadir FHA, Al-Massad FK, Moore GR (1992). Biochem J.

[86] O’Halloran TV, Culotta VC (2000). J Biol Chem.

[87] Frey AG, Nandal A, Park JH, Smith PM, Yabe T, Ryu MS, Ghosh MC, Lee J, Rouault TA, Park MH, Philpott CC (2014). Proc Natl Acad Sci.

[88] Garg RP, Vargo CJ, Cui X, Kurtz DMJ (1996). Biochemistry.

[89] Quail MA, Jordan P, Grogan JM, Butt JN, Lutz M, Thomson AJ, Andrews SC, Guest JR (1996). Biochem Biophys Res Commun.

[90] Yao H, Wang Y, Lovell S, Kumar R, Ruvinsky AM, Battaile KP, Vakser IA, Rivera M (2012). J Am Chem Soc.

[91] Wang Y, Yao H, Cheng Y, Lovell S, Battaile KP, Midaugh CR, Rivera M (2015). Biochemistry.

[92] O’Connell MJ, Ward RJ, Baum H, Treffry A, Peters TJ (1988). Biochem Soc Trans.

[93] Ward RJ, O’Connell MJ, Dickson DPE, Reid NMK, Wade VJ, Mann S, Bomford A, Peters TJ (1989). Biochim Biophys Acta.

[94] Kidane TZ, Sauble E, Linder MC (2006). Am J Physiol Cell Physiol.

[95] Asano T, Komatsu M, Yamaguchi-Iwai Y, Ishikawa F, Mizushima N, Iwai K (2011). Mol Cell Biol.

[96] Mancias JD, Wang X, Gygi SP, Harper JW, Kimmelman AC (2014). Nature.

[97] Douglas T, Ripolli DR (1998). Protein Sci.

[98] Takahashi T, Kuyucak S (2003) Biophys J 84:2256–2263 10.1016/S0006-3495(03)75031-0 PMC1302792 12668434

[99] Yao H, Rui H, Kumar R, Eshelma K, Lovell S, Battaile KP, Im W, Rivera M (2015). Biochemistry.

[100] Liu X, Jin W, Theil EC (2003). Proc Natl Acad Sci.

